# Cardiac MRI improves cardiovascular risk stratification in hazardous occupations

**DOI:** 10.1186/s12968-019-0544-5

**Published:** 2019-07-29

**Authors:** David A. Holdsworth, Iain T. Parsons, Rebecca Chamley, Joseph Britton, Christopher Pavitt, A. John Baksi, Stefan Neubauer, Joanna d’Arcy, Edward D. Nicol

**Affiliations:** 1 Royal Centre for Defence Medicine, Queen Elizabeth Hospital, Birmingham, England; 2 Oxford Heart Centre, Oxford University Hospitals NHS Foundation Trust, Oxford, England; 3 Department of Cardiology, Royal Brompton and Harefield NHS Foundation Trust, Sydney Street, London, SW36NP England; 4 Royal Berkshire NHS Foundation Trust, Reading, England; 5 Division of Cardiovascular Medicine, University of Oxford, Oxford NIHR Biomedical Research Centre, Oxford, England

**Keywords:** Occupational, Risk, Aviation medicine, Ectopy, Cardiomyopathy

## Abstract

**Background:**

The benefit of cardiovascular magnetic resonance Imaging (CMR) in assessing occupational risk is unknown. Pilots undergo frequent medical assessment for occult disease, which threatens incapacitation or distraction during flight. ECG and examination anomalies often lead to lengthy restriction, pending full investigation. CMR provides a sensitive, specific assessment of cardiac anatomy, tissue characterisation, perfusion defects and myocardial viability. We sought to determine if CMR, when added to standard care, would alter occupational outcome.

**Methods:**

A retrospective review was conducted of all personnel attending the RAF Aviation Medicine Consultation Service (AMCS) for assessment of a cardiac anomaly, over a 2-year period. Those undergoing standard of care (history, examination, exercise ECG, 24 h-Holter and transthoracic echocardiography), and those undergoing a CMR in addition, were identified. The influence of CMR upon the final decision regarding flying restriction was determined by comparing the diagnosis reached with standard of care plus CMR vs. standard of care alone.

**Results:**

Of the ~ 8000 UK military aircrew, 558 personnel were seen for cardiovascular assessment. Fifty-two underwent CMR. A normal TTE did not reliably exclude abnormalities subsequently detected by CMR. Addition of CMR resulted in an upgraded occupational status in 62% of those investigated, with 37% returning to unrestricted duties. Only 8% of referrals were undiagnosed following CMR. All these were cases of borderline chamber dilatation and reduction in systolic function in whom diagnostic uncertainty remained between physiological exercise adaptation and early cardiomyopathy.

**Conclusions:**

CMR increases the likelihood of a definitive diagnosis and of return to flying. This study supports early use of CMR in occupational assessment for high-hazard occupations.

**Electronic supplementary material:**

The online version of this article (10.1186/s12968-019-0544-5) contains supplementary material, which is available to authorized users.

## Introduction

Cardiovascular Magnetic Resonance Imaging (CMR) provides important prognostic information and aids risk stratification in the majority of cardiovascular diseases [1]. CMR is therefore a potentially useful investigation in the occupational assessment of those in high-hazard occupations. No previous study has assessed the utility of CMR in the occupational setting, with the exception of professional athletes [2, 3]. The aim of this study was to explore the impact and utility of CMR, from an occupational perspective, in military aircrew.^1^ We hypothesised that the addition of CMR would provide a more specific cardiovascular diagnosis and aid the accurate determination, or exclusion, of cardiac pathology. This enhancement to standard of care would optimise decision-making, allowing prompt, appropriate occupational restriction, or a swift return to flying duties. This has clear benefits for both the individual and the employer.

In the UK’s three Armed Services there are approximately 8000 personnel who have an occupational role as a pilot, other member of aircrew or controlling aircraft in flight. These individuals undertake exacting, often hazardous activity, during which any sudden or insidious incapacitation or distraction may have catastrophic results. For these reasons there are strict criteria defining the required medical standards and acceptable electrocardiogram (ECG) appearances in this unique cohort [4]. Periodical medical evaluation, often including a 12-lead ECG, is completed frequently, and any anomalies lead to further assessment. ECGs are recorded on appointment; at ages 25 and 30 years old; at 2-year intervals from 30 to 39 years old; annually from 40 to 49 years old; and every 6 months from the age of 50 upwards. Further investigations are typically conducted while the individual is occupationally restricted: either partially (for example, not being permitted to fly solo – pilots who are required to fly “as or with” another suitably qualified pilot) or completely (i.e. the aircrew are “grounded” and unable to fly). For example, the finding of 2 or more ventricular ectopic beats on a standard 12-lead ECG (paper speed: 25 mm/s) mandates the recording of a 24-h ECG (Holter monitor), transthoracic echocardiography (TTE) and exercise ECG testing (exercise tolerance test - ETT) which constitute the recommended first line investigations [4]. Abnormalities in any of these first-line investigations may warrant further specialist cardiovascular investigations, both anatomical and functional. These may include computed tomography (CT) coronary angiography (CTCA), dobutamine stress echo (DSE), single-photon emission computed tomography (SPECT) and CMR, which may include perfusion and viability assessment. Typical indications for requesting a CMR include the finding of a greater than 2% burden of ventricular ectopy on a 24-h ECG and an ECG pattern consistent with cardiomyopathy e.g. the appearance of left ventricular hypertrophy with strain. This approach to occupational cardiology is therefore different to that of standard clinical cardiology. Aircrew are usually asymptomatic and an abnormal ECG or (less commonly) abnormal clinical examination acts as a ‘trigger’ to investigate further and to diagnose or exclude sub-clinical, but potentially occupationally significant, cardiac pathology. The ‘rules’ determining occupational restriction are relatively simple. If the estimated risk of sudden (or insidious) incapacitation or distraction, as a result of a medical problem, is greater than 1 % per year, then flying (pilots) or controlling (air-traffic controllers) even in multi-crew environments (i.e. with a co-pilot who is suitably trained on the aircraft) is not permitted. This ‘1% rule’ is based on the fact that a 1% risk of incapacitation/distraction is estimated to equate to the catastrophic loss of an aircraft due to ‘human failure’ once in every 10^9^ h. This estimate includes assumptions about the amount of pilot operation time spent on safety critical activity (i.e. take off, landing, collision avoidance) and the likelihood of a co-pilot being able to assume full control in time to prevent an accident. One accident every 10^9^ h is the ‘acceptable’ risk threshold applied across all aviation engineering systems. Although the *rule* is simple, making an evidence-based estimate that the risk does not exceed this threshold is not. In military aviation, solo flight (e.g. piloting a high-performance fast jet) is not permitted if the medical risk of incapacitation or distraction ‘exceeds that of the individual’s peers’.

CMR provides highly accurate morphological and functional assessment with excellent reproducibility [5, 6], permitting accurate quantification, or highly sensitive exclusion, of pathology [7]. It is the gold-standard for the measurement of ventricular volumes and mass [8, 9] and is significantly more accurate and reproducible than echocardiography [5, 6], particularly for right ventricular assessment [6]. The addition of late gadolinium contrast enhancement (LGE) and T1 or T2 preparation pulses (“mapping”) provides tissue characterization that cannot be achieved by echocardiography or matched by any other imaging modality. In cases of LV dysfunction CMR can discriminate myocarditis; myocardial infarction and various forms of cardiomyopathy [10], and provides the most accurate non-ionising imaging of the great vessels [11]. There is a large body of data linking CMR to clinical outcome, which permits robust risk stratification of aircrew [1]. A number of studies have demonstrated that the presence of LGE on CMR is the most important independent predictor of major adverse cardiac events (MACE) compared to other clinical predictors, including ejection fraction (EF) [12, 13]. The absence of perfusion abnormalities on CMR also confers a good prognosis [14]. Many clinical questions arising in aircrew start with an abnormal ECG, in particular frequent ventricular ectopics, which trigger the recording of a 24-h ECG and other standard of care investigations. The triggering ECG often turns out to be a false-positive. CMR, which is not only highly sensitive but also specific, is well suited to allow swift, evidence-based assurance, in those with abnormal ECG, abnormal Holter ECG or equivocal echocardiogram. The purpose of this work was to determine the clinical occupational impact of CMR in the cardiovascular risk assessment of individuals in a high-hazard occupation.

## Methods

We conducted a retrospective review of the clinical management of military aircrew referred for cardiovascular assessment over a 2-year period (2014–2016). We included all consecutive cases in which a CMR was performed in addition to standard of care investigations at the Royal Air Force (RAF) Aviation Medicine Consultation Service (AMCS). AMCS, established in 2011, is the referral centre for all clinical occupational queries relating to aircrew in the British Armed Forces [15]. Standard care included history and examination; ETT; 24-h Holter-monitor and TTE in all cases. The following information was recorded in all cases: occupational role; clinical indication for referral; CMR results; and the occupational status prior to, and following, CMR.

All scans were performed at one of two centres: The Royal Brompton and Harefield NHS Foundation Trust, London, and the Oxford Centre for Clinical Magnetic Resonance Research, (OCMR), Oxford. Scans were performed using a conventional cardiac-enabled MRI scanner (1.5 T Avanto, Siemens Medical Solutions, Erlangen, Germany). Images were obtained using standard, published protocols [16]. Images were reported both as part of routine clinical care, and independently by an additional consultant holding SCMR Level 3 CMR accreditation. Occupational status prior to, and following, the CMR was recorded. Aircrew were occupationally graded according to standardised criteria [4], and classified in 3 categories: unrestricted flying, able to fly with restrictions, or grounded (unable to fly in an occupational role).

The effects of TTE and CMR scans on occupational outcome were evaluated using Chi-squared or Fisher’s exact tests. Statistical analysis was performed using SPSS (version 22, IBM). A *p*-value ≤0.05 (two-tailed) was taken to indicate statistical significance.

## Results

Over a two-year period, 1025 personnel were referred for clinical outpatient assessment. Referrals were made from a UK military aviation medicine population of ~ 8000 personnel. Of the 1025 medical referrals, 558 (54%) were made for evaluation of suspected cardiovascular disease [15]. Referral was by primary healthcare physicians or occupational physicians, the majority of whom are trained aeromedical examiners (AMEs). The majority of referrals (Table 1) were for ECG anomalies identified during ‘fitness to fly’ medicals. Of this cardiovascular referral group, 52/558 (9.3%) underwent a CMR scan.

**Table 1:** Indication for CMR by frequency of referral

Category^a^	Subtype/Description	CMR clinical question
Ventricular ectopy^b^	> 2% ectopy on 24 h Holter ECG following the finding of ≥2 VEs on 12 lead ECG (25 mm/s)	?cardiomyopathy/scar
	> 2% ectopy on 24 h Holter ECG performed for other reason	?cardiomyopathy/scar
ECG appearances consistent with cardiomyopathy	Pathological TWI; LVH with marked strain pattern; bundle branch block	?cardiomyopathy/scar
Structural changes on echo suggestive of possible cardiomyopathy^c^	Chamber dilatation and mild reduction in resting systolic function	?cardiomyopathy/scar
	Left or right ventricular hypertrophy/cardiomyopathy	?cardiomyopathy/scar
Chest pain	History of chest pain and elevated troponin, +/− ischaemic ECG changes	?myocardial infarction/?pattern more consistent with myocarditis/?inducible ischaemia
ECG consistent with possible coronary artery disease	Q-waves, ST segment and T-wave changes, abnormal R-wave amplitude	?myocardial infarction/?inducible ischaemia/?wall thinning/?RWMA/?ventricular aneurysm
Abnormal findings on exercise ECG stress test	ST changes; tachyarrhythmia; bundle branch block or other conduction abnormality; development of hypotension or failure to increase SBP	?cardiomyopathy/scar/?inducible ischaemia
Coronary artery disease	Previously diagnosed	LV function/?RWMA/?myocardial infarction/inducible ischaemia and viability
Bicuspid aortic valve	Previously diagnosed	?aortopathy; valve appearance/stenosis;
Other	Including: shortness of breath; pre-syncope; palpitations; family history of cardiomyopathy; CMR as part of cardiovascular work-up following another diagnosis - T2DM	?cardiomyopathy/scar/?inducible ischaemia

^a^Some individuals met more than one ‘indication category’ for CMR. Family history was a component of the total pre-test risk assessment of cardiovascular disease in several cases

^b^This includes frequent isolated VEs and the finding of couplets, NSVT and ventricular bigeminy/trigeminy

^c^NSVT: non-sustained VT; TWI: T-wave inversion; LVH: left ventricular hypertrophy; RWMA: regional wall motion abnormality; SBP: systolic blood pressure

Abbreviations: *NSVT* Non-sustained VT, *TWI* T-Wave inversion, *LVH* Left ventricular hypertrophy, *RWMA* Regional wall motion abnormality, *SBP* systolic blood pressure

For fully formatted Table [see additional file 2]

Demographic details are summarised in Table 2. The CMR cohort had a median age of 43 years (range 20–62 years) and was predominantly male (96%). The largest occupational group was pilots (35%), including multi-engine, fast jet, rotary and remotely piloted aircraft system (RPAS) operators. Air-traffic controllers and aeromedical personnel comprised 27% and represented the subset of the population who do not fly as part of their day to day role. The remainder (38%) included rear crew, flight engineers and navigators. The age and gender distribution of the pilot subgroup did not differ from that of the whole group.

**Table 2:** Age and gender of referral groups

Population	Number	Median age (interquartile range)	Male (%)
UK military aircrew	~ 8000	41 (28–52)	95.9%
AMCS cardiovascular referrals	558	46 (31–53)	94.2%
AMCS referrals sent for CMR	52	43 (33–50)	96.2%
Normal CMR	24	44 (33–51)	92.0%
Pathological or indeterminate CMR	28	43 (33.5–48)	100.0%

For fully formatted Table [see additional file 3]

**Fig. 1:**
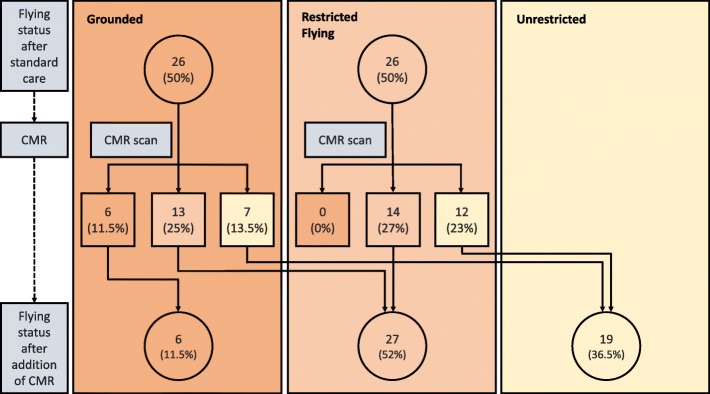
Detailed effect of CMR scan on occupational disposal (see Additional file 1)

The effect of the addition of a CMR scan to standard of care is shown in Fig. 1 and Table 3. Prior to being scanned, no one referred for CMR was flying or controlling air-traffic unrestricted on the basis of standard care, half were working with significant limitations imposed (most commonly the limitation was a requirement to work in close proximity to another equally qualified person) and the other half were grounded. Following CMR investigation, over one third of personnel (36.5%) were working unrestricted and a further 52% were able to work with occupational limitations. Only 11.5% remained unable to work at all.

**Table 3:** Occupational disposal of military aircrew before and after CMR scan

	Occupational disposal				
	Pre-CMR scan		Post-CMR scan		Change in disposal
	Number	Proportion	Number	Proportion	*p*-value
Grounded	26	50%	6	11.5%	< 0.001
Flying restriction	26	50%	27	52%	
Unrestricted flying	0	–	19	36.5%	

For fully formatted Table [see additional file 4]

The significance of change in occupational disposal before and after CMR was determined using a Chi-squared test. The terms ‘grounded’; ‘flying restriction’ and ‘unrestricted flying’, can be taken to be synonymous with ‘unable to work in role’; ‘able to work within trained role, with occupational restrictions’ and ‘unrestricted working’ for those in non-pilot roles

Table 4 presents the results of assessment by CMR both by the indication for referral and the final diagnosis after CMR. By far the commonest reason for referral was for an abnormal ECG in asymptomatic individuals (73% of referrals). One in 4 referrals (27%) were for asymptomatic ventricular ectopy on 12-lead ECG. The remaining asymptomatic ECG abnormalities together accounted for a further 46% of referrals (33% had an abnormal TTE and 13% a normal TTE). The final group of 27% of referrals comprised patients investigated for symptoms of chest pain (12%), shortness of breath, presyncope, palpitations or following diagnosis with another vascular/metabolic pathology. A small minority (6%) was investigated owing to a family history of cardiomyopathy and/or sudden cardiac death.

**Table 4:**
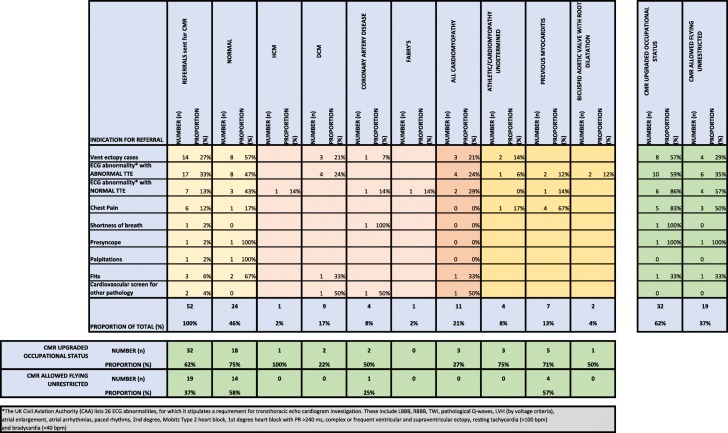
Occupational disposal by referral and diagnosis, showing the influence of CMR scan

In spite of referral for ECG abnormalities; cardiorespiratory symptoms; known FHx of cardiomyopathy or new cardiovascular pathology, 24 (46%) of military aircrew had no pathology identified on CMR. However, 11 (21%) were confirmed to have a cardiomyopathy. Most commonly, this was DCM (9 (17%) of referrals) but there was also a case of HCM, and one of Fabry’s cardiomyopathy. A further 7 (13%) were diagnosed with previous myocarditis, including two thirds of those referred with chest pain. 4 (8%) were diagnosed with coronary artery disease. One had suffered a ‘silent’ infarct, with basal inferior sub-endocardial scar on LGE imaging and corresponding findings on myocardial perfusion scan. Two patients had intermediate severity coronary disease, detected on CTCA (CT coronary angiography) and remain under periodic follow up to proactively detect the development of inducible ischaemia. The fourth individual was found to have a blocked right coronary artery with good collateralisation and a myocardial ischaemic burden < 10%.

In just 4 (8%) of aircrew it was not possible to make a definite diagnosis. In these cases, diagnostic uncertainty remained between physiological exercise adaptation or early cardiomyopathy. Further imaging, following a period of 3 months detraining, was recommended. In cases of individuals diagnosed as ‘normal’ on CMR who were unable to fly in any capacity, this was most commonly due to ongoing ventricular ectopy.

The referral indication most strongly associated with pathology was chest pain, with only one case judged to be normal and 4 (67%) diagnosed with previous myocarditis. A referral with an abnormal 12-lead ECG was associated with a post-CMR ‘normal’ diagnosis in 11 (46%) of cases. An abnormal ECG with a ‘normal’ TTE did not increase the likelihood of a ‘normal’ CMR diagnosis, when compared to abnormal ECG and an ‘abnormal’ TTE (43% vs. 47%, *p* = 1.0). The other common referral pathway, ventricular ectopy (> 1 VE per 12-lead ECG trace triggers a 24 h Holter ECG), was associated with a ‘normal’ diagnosis in 8 (57%) of cases, but with only 4 (29%) returning to unrestricted flying. In most cases this was because of the mandatory occupational restriction. Aircrew are investigated when the burden of ventricular ectopy exceeds 2%. Aircrew cannot operate independently (‘solo’ pilot) with a ventricular ectopy burden > 7.5%. Ventricular ectopic burden > 10% demands a recommendation of ‘grounding’.

Table 5 lists the CMR parameters of this cohort divided into normal, DCM, HCM and undetermined (physiological athletic adaptation or early cardiomyopathy), alongside published normal values. The prevalence of HCM and DCM are listed alongside the up to date published prevalence in a developed world population.

**Table 5:** – CMR chamber dimensions for military aircrew cohort

Diagnosis	Mean Indexed LVEDV (ml/m^2^)	Mean Indexed LVESV (ml/m2)	LVEF %	Mean LV wall thickness^a^ (mm)	Mean Mass Index (g/m2)	Prevalence in aircrew population^b^	Published prevalence
Normal aircrew population^c^	82	29	66	82	65		
Normal men < 60 years old [9]	82	28	66	–	74		
Athletic / Cardiomyopathy	102	40	61	101	82	0.05%	
Dilated Cardiomyopathy	111	55	51	93	72	0.11%	0.4% [17]
Hypertrophic Cardiomyopathy	75	26	71	103	95	0.013%	0.2% [18]

Mean LV volumes, ejection fraction, wall thickness and mass index for cardiomyopathy diagnoses in comparison to normal scans

^a^Mean wall thickness calculated from mean of anterior, lateral, inferior and septal walls at basal, mid and apical levels

^b^Population at risk (PAR) defined as all aircrew (*n* = 8000) PAR defined as all aircrew (*n* ≈ 8000),

^c^Data are taken from the current study cohort found to be normal on CMR

For fully formatted Table [see additional file 5]

## Discussion

These data demonstrate that the addition of CMR to the Royal Air Force standard of care cardiovascular assessment substantially increases the likelihood of a return to flying. In the majority of this cohort, cardiovascular assessment was triggered by an ECG abnormality. In this setting, a normal TTE did not alter the likelihood of a pathological diagnosis on CMR. Therefore, a normal echocardiogram does not provide sufficient reassurance in the high-hazard setting. In the cases referred for CMR, Royal Air Force cardiologists were likely to recommend restricted flying, including grounding in some cases, pending the scan result. This reflects the high index of residual clinical suspicion following standard of care investigations. Without the addition of CMR, these aircrew would have remained ‘grounded’ or received much more stringent occupational restriction on their flying or controlling duties.

CMR was found to be normal in 46% of our cohort. 58% of this “normal” group were able to return to full flying duties with a further 17% of the group able to fly in a restricted capacity. The reason for ongoing restriction in the remainder was typically ongoing ventricular ectopy. Fast jet aircrew are exposed to significant positive sustained acceleration (+G_z_: directed from head to foot), which causes venous pooling of blood in the legs and decreased cerebral perfusion. They are usually not permitted to fly as solo pilots with arrhythmia. Positive G (+G_z_), which may briefly reach 15–20 times the acceleration due to earth’s gravity, is arrhythmogenic and antiarrhythmic medications such as beta-blockers are incompatible with high performance flying due to the need to mount an appropriate tachycardia when subjected to sustained acceleration [19].

Whilst the decision to return to flying duties is based on a combination of clinical history, examination, cardiac investigations, requirement for medication and an individual’s flying role, CMR frequently provided critical information, which facilitated less restrictive management and allowed a safe-return to flying.

The high sensitivity and high specificity of CMR are both beneficial for occupational risk management in a high-hazard setting. High sensitivity (low false-negative rate) means that a high degree of confidence can be placed in a ‘normal’ diagnosis from CMR, relevant to both structural heart disease and coronary artery disease, in the case of perfusion CMR. The high specificity (low false positive rate) is valuable in terms of the confidence attributed to a positive diagnosis of disease. However, given the huge resources (money and time) invested in the highly-trained individuals concerned, the main strength of highly specific diagnostic testing is in reducing the rate of career-ending medical decisions based on misdiagnosis.

Even in this small cohort, the potential financial saving to the military is enormous. A fully trained pilot costs in excess of £1 M to train [20], and 8 grounded pilots were returned to flying duties following CMR during this 2-year period alone. CMR scans have a higher tariff than echocardiograms (£468.74 for an NHS outpatient contrast CMR and £70.40 for an NHS outpatient transthoracic echo in patients aged 19 years and older), and CMR is not always suitable for first-line use. However, given the opportunity costs of grounding aircrew, following years of training and experience, in selected patients CMR is a highly cost-efficient investigation.

CMR must be interpreted within the clinical context and will not provide a definitive answer in all cases. Myocarditis can be a challenging diagnosis to confirm with CMR. Using the Lake Louise criteria with T2 STIR, early and late gadolinium enhancement sequences, myocarditis may be diagnosed with 76% sensitivity and 96% specificity [21]. In our cohort, this allowed a confident diagnosis of myocarditis in 7 personnel (13%). Of this small group, 4 returned to unrestricted flying.

Only 4 cases (8%) had non-diagnostic CMR findings. These were cases in which it was not possible to distinguish between cardiac remodelling associated with physical training and early cardiomyopathy. Differentiating between physiological left ventricular hypertrophy secondary to exercise and hypertrophic cardiomyopathy is a recognised problem [22], particularly in early disease when no LGE is present. The LARGE heart study demonstrated, using pre and post CMR scans, the development of asymmetrical hypertrophy in ~ 500 young male military recruits during a 12-week period of intense exercise training (average energy expenditure 5000 Calories per day) associated with an increased prevalence of asymmetrical septal hypertrophy from 2.2 to 10% [20]. Several strategies have been proposed to attempt to differentiate cardiomyopathy from athlete’s heart, such as measuring diastolic wall thickness to volume ratio [23], or measurement of peak oxygen uptake on cardiopulmonary exercise testing [24]. Nevertheless, distinguishing athletic cardiac adaptation from cardiomyopathy remains a clinical challenge. This issue is further complicated by the results of a recent study showing that patients who undergo even a moderate amount of physical activity have cardiac remodelling on CMR [25], as well as by evidence suggesting that physiological athletic adaptation of the heart may not always be benign [14]. Newer CMR sequences are likely to assist in differentiating these groups. These sequences include quantification of the extracellular volume with T1 mapping [26, 27], which may reduce the requirement for repeated scans and follow-up so reducing uncertainty and further shortening the time to definitive diagnosis and occupational decision making.

Within the group of 8000 British Forces aviation personnel under regular surveillance, the prevalence of DCM (0.11%) is higher than the traditionally reported figure of 0.04% [28]. However, recent estimates of DCM prevalence in a developed population are 10-fold higher at 0.4% [17]. It has been proposed that 0.4% is the true prevalence based on accessible healthcare and advanced diagnostic modalities, such as CMR [17]. The prevalence of HCM in our cohort is 0.013%, more than 10-fold lower than the reported prevalence of 0.2% [18]. The lower prevalence of hypertrophic cardiomyopathy is likely to reflect three levels of screening, which are intended to prevent recruitment of individuals with significant cardiovascular disease. First, at recruit medicals, an applicant will be asked about a family history of unexplained or sudden cardiac death; a family history of heart failure, or requirement for heart transplant; and any personal cardiac history. Second, the potential recruit undergoes physical examination. Finally, all service personnel who will undertake safety-critical functions in aviation will have an ECG. At any of these stages, a positive finding would preclude recruitment/training. In fact, given the degree of scrutiny applied in the aviation recruitment process, the finding of cardiomyopathy in 21% of referrals is intriguing. Many cardiomyopathy phenotypes do not develop until young adulthood or middle age. Aircrew continue to undergo regular, increasingly frequent, periodic medical examination and 12-lead ECG recordings throughout their service life. Given this ongoing focussed medical attention, and the frequent (9%) use of CMR as part of the cardiovascular assessment, the continued finding of cardiomyopathy in aircrew is perhaps not unexpected.

## Limitations

This is a retrospective, single country study of a highly selected group of predominantly male military aircrew. By definition each patient serves as their own control (standard of care (before CMR) vs. standard of care plus CMR). This study is subject to the bias associated with non-randomised studies and retrospective analyses. As the scans were carried out at two regional centres, small protocol variance may introduce further variation, although both centres followed standard CMR protocols [16]. There is some heterogeneity in aircrew roles, e.g. the physiological challenges to a fast-jet pilot are different to those of an air-traffic controller, so occupational management may not always be applied uniformly. A randomised, prospective study was not possible, due to the ethical challenges inherent in occupational care of a group engaged in hazardous work. Clinical equipoise does not exist between standard care and standard care plus CMR, given the clear advantages of CMR to the individual, and to flight safety, in many cases. Though there are limitations in extrapolation of non-randomised data, these findings, from a real-world military aviation population of 8000, illuminate the significant benefit of CMR in this previously unstudied area of occupational cardiology.

## Conclusion

CMR is an essential investigation in the occupational assessment of military aircrew with potential cardiovascular disease. A normal TTE does not reliably exclude pathology in this cohort. The high-sensitivity of CMR permits confident exclusion of pathology and the high-specificity reduces the number of key personnel restricted unnecessarily. As a result, CMR expedites decision making and substantially reduces costly occupational restriction of military aircrew. These benefits of CMR may reasonably be expected to also apply to the occupational management of other high-hazard occupations.

## Additional files

Additional file 1: **Figure S1** – Detailed effect of CMR scan on occupational disposal. (PPTX 68 kb)

Additional file 2: **Table S1** – Indication for CMR by frequency of referral. (XLSX 13 kb)

Additional file 3: **Table S2** – Age and gender of referral groups. (XLSX 9 kb)

Additional file 4: **Table S3** – Occupational disposal of military aircrew before and after CMR scan. (XLSX 9 kb)

Additional file 5: **Table S5** – CMR chamber dimensions for military aircrew cohort. (XLSX 9 kb)

## Data Availability

The datasets generated and/or analysed during this study are not publicly available, as they pertain to the British Military Aviation population, but are available from the corresponding author on reasonable request.

## Abbreviations

AMCS: Aviation Medicine Consultation Service
CMR: Cardiovascular Magnetic Resonance
CTCA: Computed tomography coronary angiography
DCM: Dilated cardiomyopathy
DSE: Dobutamine stress echo
ECG: Electrocardiogram
ETT: Exercise Tolerance Testing
HCM: Hypertrophic cardiomyopathy
LGE: Late Gadolinium enhancement
RAF: Royal Air Force
RPAS: Remotely Piloted Aircraft System
SPECT: Single-photon emission computed tomography
TTE: Transthoracic echocardiography

## Footnotes

1 “Military aircrew” incorporates all flight-safety critical personnel: pilots, non-pilot aircrew and air-traffic controllers.
