# Supplementary material for: Cardiac MRI improves cardiovascular risk stratification in hazardous occupations
Source: J Cardiovasc Magn Reson. 2019 Jul 29;21:48. doi: 10.1186/s12968-019-0544-5 (PMC6661777; doi:10.1186/s12968-019-0544-5)
Supplement: Supplementary file 1 — Figure S1 – Detailed effect of CMR scan on occupational disposal. (PPTX 68 kb) [file 12968_2019_544_MOESM1_ESM.pptx]

## Slide 1
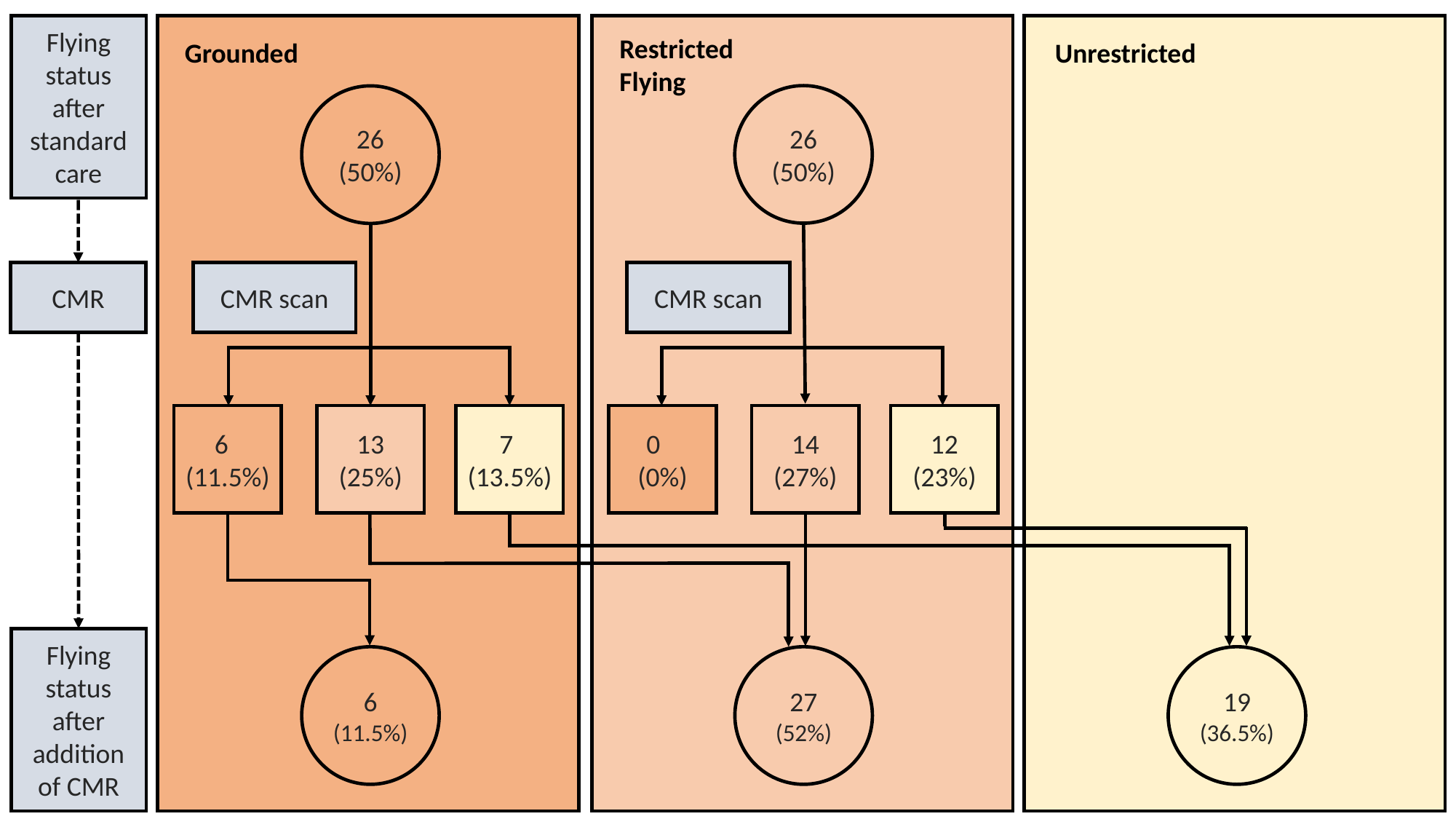

Flying status after standard care
Gr
Restricted Flying
Grounded
Unrestricted
26 (50%)
26
(50%)
CMR
CMR scan
CMR scan
6 (11.5%)
13 (25%)
7 (13.5%)
0 (0%)
14 (27%)
12 (23%)
Flying status after addition of CMR
6
(11.5%)
27 (52%)
19 (36.5%)
